# O-Glycosylation Changes in Serum Immunoglobulin G Are Associated with Inflammation Development in Advanced Endometriosis

**DOI:** 10.3390/ijms23158087

**Published:** 2022-07-22

**Authors:** Katarzyna Sołkiewicz, Monika Kacperczyk, Hubert Krotkiewski, Marcin Jędryka, Ewa Maria Kratz

**Affiliations:** 1 Department of Laboratory Diagnostics, Division of Laboratory Diagnostics, Faculty of Pharmacy, Wroclaw Medical University, Borowska Street 211A, 50-556 Wroclaw, Poland; monika.kacperczyk@umw.edu.pl; 2 Hirszfeld Institute of Immunology and Experimental Therapy, Polish Academy of Sciences, Weigla 12, 53-114 Wroclaw, Poland; krotkiew@iitd.pan.wroc.pl; 3 Department of Oncology, Gynecological Oncology Clinic, Faculty of Medicine, Wroclaw Medical University, Hirszfeld Square 12, 53-413 Wroclaw, Poland; marcin.jedryka@umw.edu.pl; 4 Department of Oncological Gynecology, Wroclaw Comprehensive Cancer Center, Hirszfeld Square 12, 53-413 Wroclaw, Poland

**Keywords:** O-glycosylation of serum IgG, multi-antennary N-glycans in IgG, lectin-ELISA, advanced endometriosis, inflammation

## Abstract

Endometriosis is a gynecological disease, the pathogenesis of which seems to be directly related to inflammatory processes with an immune basis. Our study aimed to analyze the O-glycosylation of native serum IgG and IgG isolated from sera of women with advanced endometriosis, without endometriosis but with benign gynecological diseases, and from a control group of healthy women, in the context of its utility for differentiation of advanced endometriosis from the other two groups of women studied. For the analysis of serum IgG O-glycosylation and the expression of multi-antennary N-glycans, lectin-ELISA with lectins specific to O-glycans (MPL, VVL, and Jacalin) and highly branched N-glycans (PHA-L) was used. The relative reactivities of isolated serum IgG O-linked glycans with specific lectins as well as the MPL/VVL O-glycosylation ratio were significantly higher in patients with advanced endometriosis and those with other gynecological diseases when compared to the control group of healthy women. We also showed significantly higher expression of PHA-L-reactive multi-antennary N-glycans in isolated IgG in the advanced endometriosis and the non-endometriosis groups in comparison to the control group. Additionally, significantly higher expression of Jacalin-reactive O-glycans in isolated IgG was observed in the non-endometriosis than in the advanced endometriosis group. The results of the ROC curve and cluster analysis additionally confirmed that the lectin-based analysis of isolated serum IgG O-glycosylation and the expression of highly branched N-glycans may help distinguish women with advanced endometriosis from healthy women. Moreover, the analysis of the expression of Jacalin-reactive i-IgG O-glycans may be helpful in differentiation between women with advanced endometriosis and patients with other gynecological diseases with an inflammatory background. In the case of non-endometriosis patients, the observed differences were most probably caused by increased expression of core 3 type O-glycans.

## 1. Introduction

Endometriosis is an inflammatory disease caused by the dissemination and proliferation of the endometrial glands outside the uterine cavity. The most common sites of occurrence of ectopic endometrial tissue are the pelvic peritoneum and pelvic organs. Moreover, endometrial ectopic tissue may also occur in organs and tissues far outside of the pelvis. The incidence of endometriosis has been estimated to affect about 10% of women of reproductive age [1].

Despite many studies, there is still a lack of non-invasive diagnostic markers of endometriosis that could indicate the development of the disease and facilitate its diagnosis and treatment. Although chronic inflammation and high estrogen concentrations are well-established characteristics of endometriosis, the etiology of this disease remains unclear. One of the theories that could explain the development of the disease is that an alteration in the immune system in terms of immune-cell recruitment, cell adhesion, and upregulation of inflammatory processes can facilitate the implantation and survival of endometriotic lesions [2,3]. 

Immunoglobulins (Igs) are glycoproteins secreted by lymphocytes B during an adaptive immune response. Their characteristic glycosylation patterns may differ in number, type, and location of oligosaccharides within each Ig isotype and subclass. Sugars play specific structural roles by maintaining and modulating Igs effector functions. Abnormal glycosylation may contribute to the development of many diseases. Among immunoglobulins, immunoglobulin G (IgG) is one of the most abundant proteins found in the blood serum of healthy subjects, which accounts for approximately 10–20% of all blood serum proteins [4]. 

Glycosylation, an enzymatic process catalyzed by a variety of glycosyltransferases and glycosidases, is a post-translational modification of a protein that links saccharides with proteins and may be regulated by a range of B cell stimuli, including environmental factors, such as stress, age or disease [5]. This versatile posttranslational modification influences proteins’ biological activity, their conformation, and the biological behavior of cells, including adhesion, molecule trafficking and clearance, receptor activation, signal transduction, endocytosis, and the interaction between a cell and its environment [6,7,8], including immunological and infectious disorders [7,9,10]. 

N-glycosylation is the most known and well-described type of glycosylation, observed in all human IgG subclasses, where the carbohydrate groups are attached to asparagine 297 (Asn 297) in the IgG CH2 domain. The N-glycans at this site can influence antibody stability [11], binding to FcγRs and complement [12], consequently modulating effector functions, such as antibody-dependent cellular cytotoxicity (ADCC) and complement-dependent cytotoxicity (CDC) [13,14,15,16]. Structurally, human IgG N-linked glycans are typically biantennary complexes, but about 10% of endogenous human serum IgG glycoforms have intersecting GlcNAc residues. The presence of an additional GlcNAc residue increases the binding affinity to FcγRIIIa, resulting in a 10–30-fold higher ADCC activity [17]. The second N-glycosylation site is found in the VH and VL (heavy and light chain of variable regions, respectively) and has been observed in 15–25% of all serum IgG. The presence of glycans in the IgG Fab region may contribute to higher antibody stability [18] and modulate antigen binding. Human IgG3 activates complement and FcγR-mediated functions more effectively than any other immunoglobulin G subclasses [19,20]. Moreover, for IgG3, apart from N-glycans present in the Fab and Fc regions, the presence of O-linked glycans in the hinge region was also observed.

The O-GalNAc glycosylation of proteins is a multi-step process in which monosaccharides are sequentially added to a growing oligosaccharide chain, which results in the formation of a core glycan. Next, the core glycan can be branched, elongated, and finally capped with terminal glycans. The simplest O-glycan is composed of GalNAc residue linked to a serine or threonine, or less frequently to a tyrosine. The initial GalNAc residue can be elongated and branched to create eight distinct O-glycan core structures, of which cores 1, 2, 3 and 4 are the most common [21]. The core 1 structure is found on all cells and abundant on circulatory glycoproteins produced by the liver [21]. The distribution of core 2, core 3, and core 4 structures is more selective, and they are predominantly found on secreted and membrane-bound proteins from epithelial cells covering external and internal surfaces [22]. The major O-glycan cores are often elongated with longer and sometimes more complex structures shared among the different types of carbohydrates, such as e.g., N-linked glycans. Elongation primarily involves N-acetyllactosamine (LacNAc type 2 chain Galβ1-4GlcNAc), often as repeating disaccharides (PolyLacNAc) and branches (Galβ1-4GlcNAcβ1-3[Galβ1-4GlcNAcβ1-6]Galβ1-4GlcNAc), although elongation with type 1 chains (Galβ1–3GlcNAc) is also possible. The O-glycan structures are finally terminated by the addition of sialic acids, fucose, or blood type antigens (ABO and Lewis structures), which protect proteins from degradation and immune recognition, or in other situations serve as important recognition motifs for carbohydrate-binding proteins involved in some biological functions such as extravasation of immune cells to sites of inflammation [23]. 

In blood serum, about 10% of IgG3 polyclonal antibodies and about 13% of IgG3 monoclonal antibodies are considered to contain O-glycans. Each IgG3 molecule can contain up to three O-glycans linked to threonine residues in the triple repeat regions within the hinge region [24]. The long hinge region of IgG3 has a high degree of surface availability, which may facilitate access and detection of the O-glycans present in this region [25]. Although the function of IgG O-glycosylation is still not fully understood, the structure of the hinge region is hypothesized to be able to protect the immunoglobulin from proteolytic cleavage, and may also help maintain the extended conformation and flexibility of IgG3 [24]. 

O-glycosylation, like N-glycosylation, is a post-translational modification that occurs after protein synthesis and consists in attaching a sugar molecule to the oxygen atom of serine (Ser) or threonine (Thr) residues of the polypeptide chain [26,27]. Mucin-type (GalNAc type) glycosylation is the best-known type of protein O-glycosylation because of its high abundance in mucins. It is a diverse form of post-translational modification, can occur in any protein, and is initiated by the family of up to 20 GalNAc polypeptide transferases that decorate proteins with GalNAc residues (GalNAcα1-O-Ser/Thr, named Tn-antigen) [21,28]. Tn-antigen is the initial step in the O-glycosylation pathway and can be further elongated in three different ways: (1) by addition of α2,6 sialic acid (formation of sialyl-Tn-antigen), (2) by addition of galactose and formation of the oligosaccharide structure Galβ1,3GalNAcα-Ser/Thr, called T-antigen (core 1), or (3) by addition of galactose to N-acetylglucosamine (core 3) [21]. Changes in immunoglobulin glycosylation, especially in its degree, have been associated with many pathological processes, including disorders in cell adhesion, tissue development, angiogenesis, fertilization, malignancy, and tumor metastasis, as well as autoimmune diseases [9,21,29,30,31,32,33,34,35,36,37,38]. 

The first goal of our research was to check whether O-glycans are expressed in serum IgG in advanced endometriosis. The present study also aimed to investigate whether the profile and degree of serum IgG O-glycosylation (both for isolated serum IgG, i-IgG, and native serum IgG, s-IgG) is characteristic of an advanced stage of endometriosis and could become a diagnostic marker supporting the diagnosis of this disease, also allowing for the differentiation of advanced endometriosis from other gynecological diseases with accompanying inflammation. IgG O-glycosylation was analyzed using a modified lectin-ELISA method, as previously described [39,40], with specific biotinylated lectins reacting primarily with complete (*Maclura pomifera* lectin and Jacalin lectin) and truncated (*Vicia villosa* lectin) O-glycans. 

We were also interested in whether, additionally to the presence of biantennary N-glycans, there are also highly branched N-glycans in IgG, and if so, whether the degree of their expression is characteristic of advanced endometriosis. The analysis was performed using the lectin-ELISA test with biotinylated *Phaseolus vulgaris* leucoagglutinin, which reacts specifically with multi-antennary N-glycans. This analysis aimed to answer whether the degree of expression of multi-antennary N-glycans in serum IgG makes it possible to differentiate advanced endometriosis from other gynecological inflammatory diseases. Another aspect of our research was to compare the results of analyses obtained for IgG isolated from serum and its native form, without prior immunoglobulin isolation. We wanted to check whether the glycosylation analysis of native serum IgG, without its prior isolation, would be sufficient to answer the above questions.

## 2. Results

**Figure 1:**
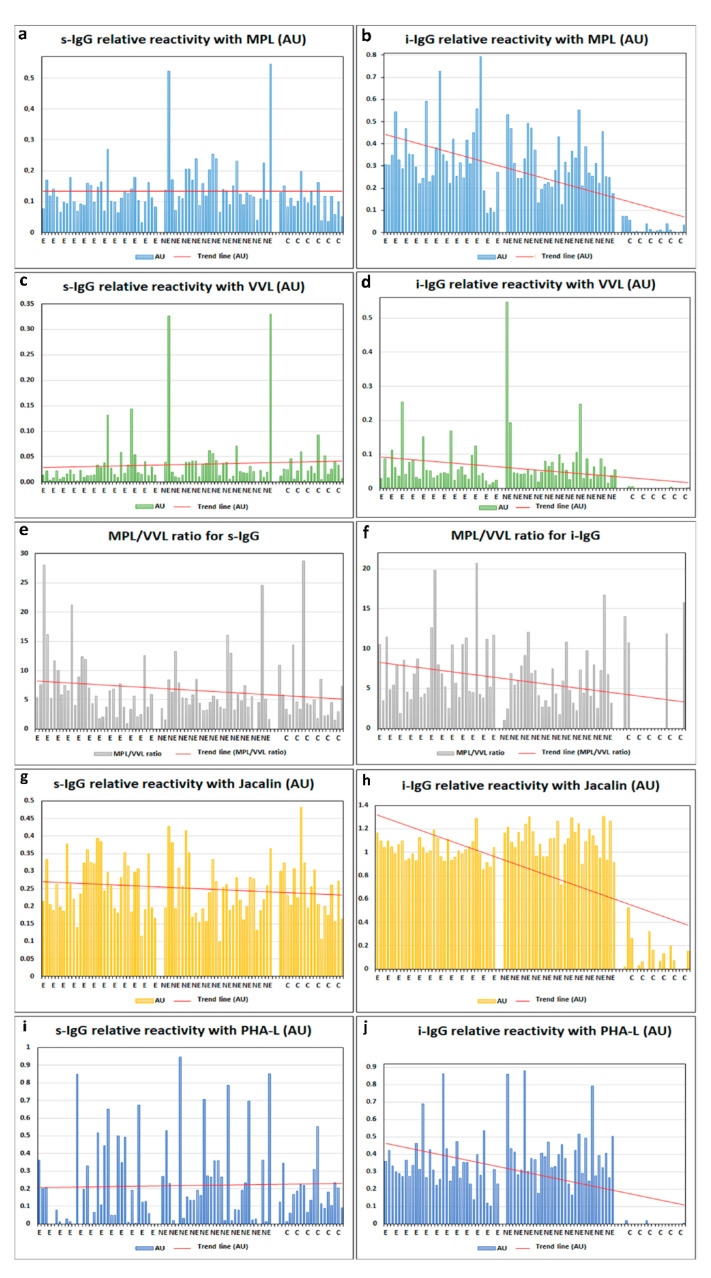
Relative reactivities of s-IgG and i-IgG glycans with specific lectins (**a**–**j**). MPL—*Maclura pomifera* lectin, VVL—*Vicia villosa* lectin, MPL/VVL—values of the ratio, Jacalin—*Artocarpus integrifolia* lectin, and PHA-L—*Phaseolus vulgaris* leucoagglutinin. For specificity of lectins see Materials and Methods section. E—advanced endometriosis, NE—non-endometriosis, and C—control group of healthy women. The trend line has been marked in red.

The relative reactivities of s-IgG and i-IgG glycans with lectins used are presented in Table 1 as mean absorbance values and standard deviations (SD) for each analyzed group, respectively. The values of relative IgG reactivities with used lectins and trend lines, measured for E, NE, and the control group of healthy women, are shown in Figure 1. To see whether the ratio between the complete MPL-reactive and truncated VVL-reactive IgG O-glycans (T-antigen and Tn-antigen, respectively) could be of importance in differentiating the group with advanced endometriosis from the rest of the study groups, the MPL/VVL ratio was calculated based on IgG relative reactivity with these two lectins (Table 1).

**Figure 2:**
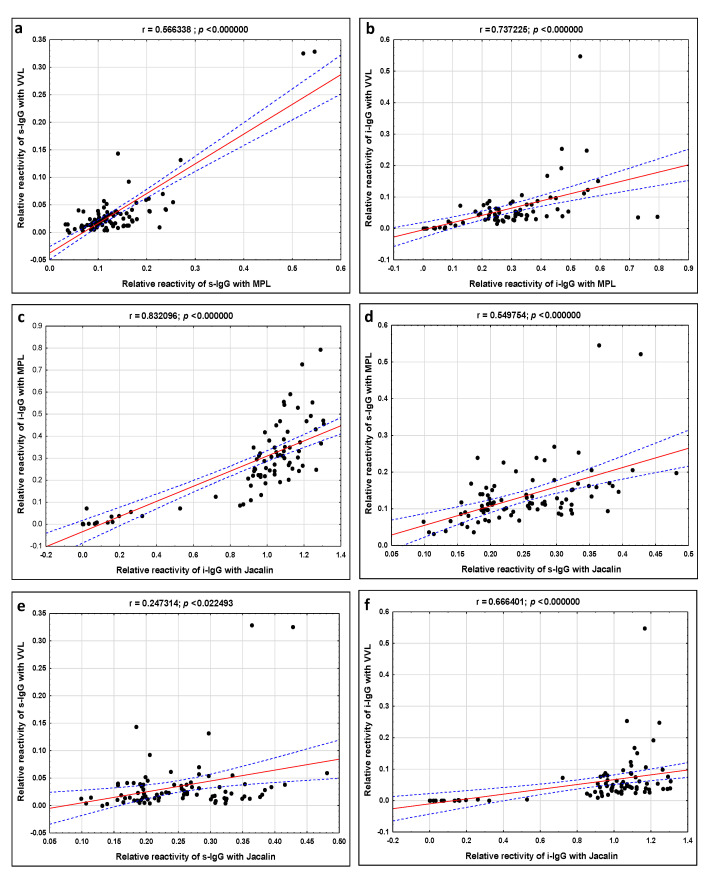
Correlations between relative reactivities of s-IgG and i-IgG O-glycans with specific lectins (**a**–**f**). Correlations were estimated according to a Spearman test, and a two-tailed *p*-value of less than 0.05 was considered significant. The 95% confidence interval is marked by dotted blue lines. MPL—*Maclura pomifera* lectin, VVL—*Vicia villosa* lectin and Jacalin—*Artocarpus integrifolia* lectin. For specificity of lectins see Materials and Methods section.

The relative reactivities of s-IgG and i-IgG glycans were presented in Table 1. The relative reactivities of MPL with s-IgG O-glycans were significantly higher in the NE group (0.169 ± 0.111 AU, median 0.136) when compared to advanced endometriosis patients (0.118 ± 0.045 AU, median 0.108 AU; *p* = 0.022764) and the control group (0.103 ± 0.042 AU, median 0.102 AU; *p* = 0.005500). The relative reactivities of s-IgG O-glycans with VVL, values of the MPL/VVL ratio, reactivities with Jacalin and reactivities of s-IgG N-glycans with PHA-L were not significantly different between the studied groups (*p* > 0.05). The Spearman rank correlations, analyzed between relative reactivities of all lectins used with s-IgG glycans, are shown in Table 2 and Figure 2. Positive moderate correlations between relative reactivities of s-IgG O-glycans with MPL vs. VVL, and MPL vs. Jacalin (r = 0.566, *p* = 0.000000; r = 0.549, *p* = 0.000000, respectively) were observed.

The relative reactivities of i-IgG O-glycans with MPL in the E (0.342 ± 0.160 AU, median 0.311) and NE (0.310 ± 0.115 AU, median 0.275 AU) groups were significantly higher (*p* = 0.000000; *p* = 0.000000, respectively) when compared to the healthy women (0.020 ± 0.025 AU, median 0.007 AU). Relative reactivities of i-IgG O-glycans with VVL in the E and NE groups were significantly higher (0.061 ± 0.051 AU, median 0.043 AU; *p* = 0.000000 and 0.080 ± 0.097 AU, median 0.054 AU; *p* = 0.000000, respectively) than those observed for the control group (0.001 ± 0.002 AU, median 0.000 AU). The values of the MPL/VVL ratio were significantly lower in the control group (2.750 ± 5.546, median 0.000) than in E (7.465 ± 4.439, median 5.530; *p* = 0.000000) and NE groups (5.885 ± 3.383, median 5.685; *p* = 0.000317). The relative reactivities of i-IgG O-glycans with Jacalin in E and NE groups were significantly higher (1.025 ± 0.0.094 AU, median 1.018 AU; *p* = 0.000000 and 1.103 ± 0.1138 AU, median 1.116 AU; *p* = 0.000000, respectively) than those observed for the control group (0.106 ± 0.140 AU, median 0.064 AU). Additionally, the expression of Jacalin-reactive i-IgG O-glycans was significantly lower in the NE than the E group (*p* = 0.006401). The relative reactivities of i-IgG N-glycans with PHA-L in the E (0.340 ± 0.148 AU, median 0.314) and NE (0.402 ± 0.169 AU, median 0.381 AU) groups were significantly higher (*p* = 0.000000; *p* = 0.000000, respectively) when compared to the control group, in which the absorbances obtained were near zero (0.002 ± 0.006 AU, median 0.000 AU). Moreover, strong positive correlations were observed between the relative reactivities of i-IgG O-glycans with MPL vs. VVL and MPL vs. Jacalin (r = 0.737; *p* = 0.00000 and r = 0.832; *p* = 0.00000, respectively). Additionally, moderate positive correlations were observed between the relative reactivities of i-IgG O-glycans with VVL and Jacalin (r = 0.666; *p* = 0.000000) (Table 2; Figure 2). There were significant but weak correlations between s-IgG and i-IgG in relative reactivities with lectins specific to O-glycans (Table 3).

### 2.1. ROC Curve Analysis

**Figure 3:**
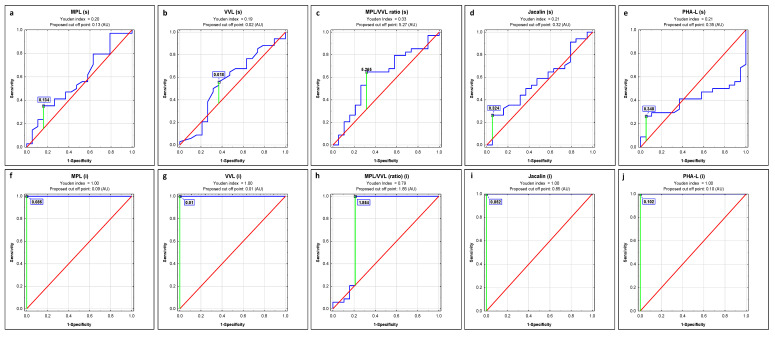
ROC curve analysis of s-IgG and i-IgG glycans’ relative reactivities with lectins for women with advanced endometriosis and healthy subjects (**a**–**j**). The reference line is marked in red, the receiver operating characteristics for the parameter in blue, and the cut-off point in green, MPL—*Maclura pomifera* lectin, VVL—*Vicia villosa* lectin, MPL/VVL—O-glycosylation ratio, Jacalin—*Artocarpus integrifolia* lectin and PHA-L—*Phaseolus vulgaris* leucoagglutinin. For specificity of lectins see Materials and Methods section.

**Figure 4:**
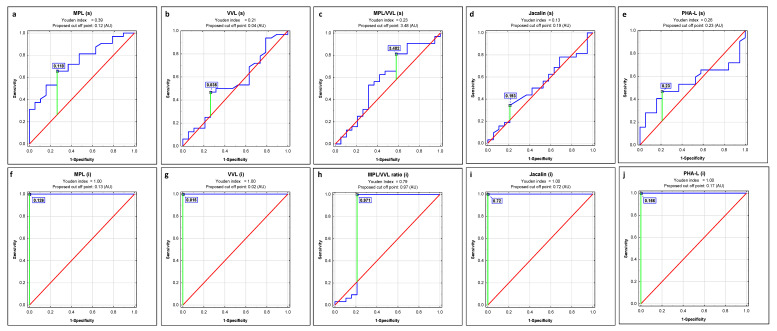
ROC curve analysis of s-IgG and i-IgG glycans’ relative reactivities with lectins for non-endometriosis women and healthy subjects (**a**–**j**). The reference line is marked in red, the receiver operating characteristics for the parameter in blue, and the cut-off point in green, MPL—*Maclura pomifera* lectin, VVL—*Vicia villosa* lectin, MPL/VVL—O-glycosylation ratio, Jacalin—*Artocarpus integrifolia* lectin and PHA-L— *Phaseolus vulgaris* leucoagglutinin. For specificity of lectins see Materials and Methods section.

ROC curve analysis of s-IgG and i-IgG glycans’ relative reactivities with all four examined lectins and the values of MPL/VVL O-glycosylation ratio in advanced endometriosis and non-endometriosis patients versus a control group of healthy women identified parameters with a sensitivity and specificity shown in Figure 3, Table 4 and Figure 4, Table 5, respectively. The Youden index method was used for the determination of cut-off points. The verification of laboratory test clinical value was based on AUC value and can be defined as 0–0.5—zero, 0.5–0.7—limited, 0.7–0.9—moderate, and >0.9 high [41].

### 2.2. Cluster Analysis

**Figure 5:**
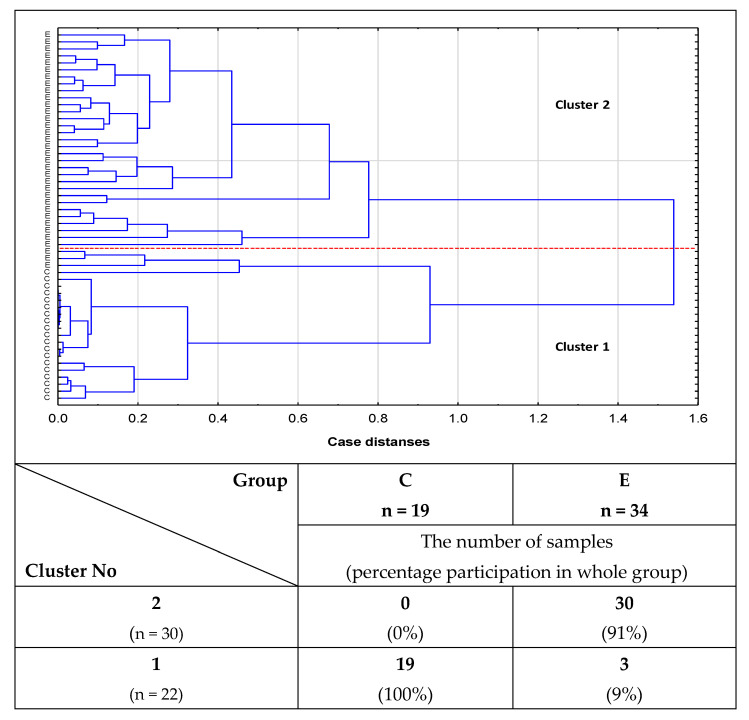
Dendrogram of cluster analysis for relative reactivities of isolated IgG with lectins in women with advanced endometriosis and healthy subjects. Cluster analysis was performed only for parameters for which the AUC value was high (>0.9) in ROC curve analysis, and which significantly differentiated patients with advanced endometriosis from healthy women. Each sample is represented by a vector of four features: MPL, VVL, Jacalin, and PHA-L relative reactivities with i-IgG glycans. E—patients with advanced endometriosis, C—control group of healthy women. MPL—*Maclura pomifera* lectin, VVL—*Vicia villosa* lectin, Jacalin—*Artocarpus integrifolia* lectin and PHA-L—*Phaseolus vulgaris* leucoagglutinin.

**Figure 6:**
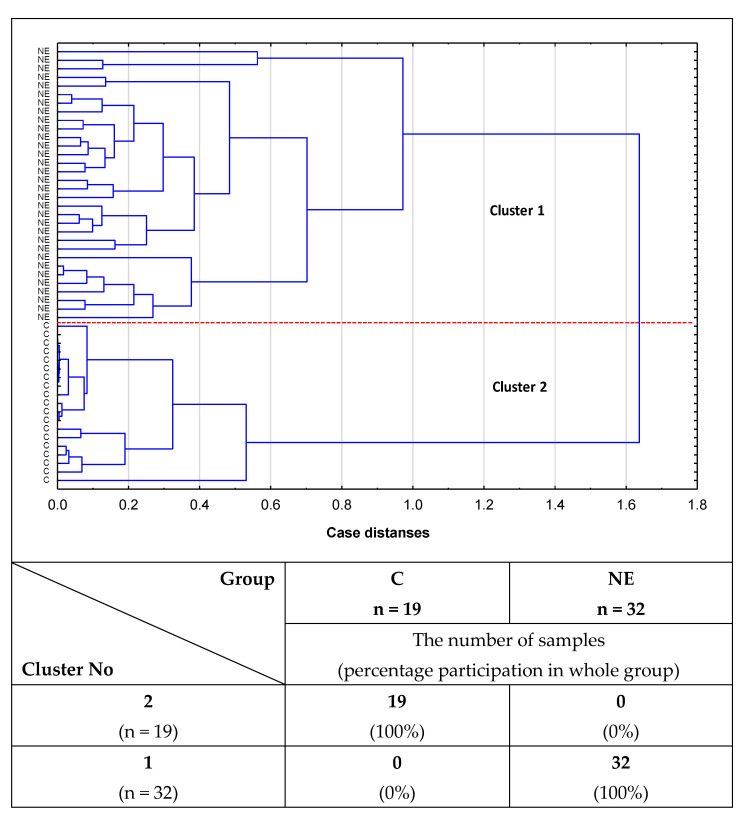
Dendrogram of cluster analysis for relative reactivities of isolated IgG with lectins in non-endometriosis women and healthy subjects. Cluster analysis was done only for parameters for which the AUC value was high (>0.9) in ROC curve analysis, and which significantly differentiated non-endometriosis patients from healthy women. Each sample is represented by a vector of four features: MPL, VVL, Jacalin, and PHA-L relative reactivities with i-IgG glycans. NE—non-endometriosis patients, C—control group of healthy women. MPL—*Maclura pomifera* lectin, VVL—*Vicia villosa* lectin, Jacalin—*Artocarpus integrifolia* lectin, and PHA-L*— Phaseolus vulgaris* leucoagglutinin.

The utility of the values of the relative reactivities of s-IgG with specific lectins for differentiation between study groups was analyzed by cluster analysis. However, due to their low clinical value, the data are not shown. The usefulness of the values of relative reactivity of i-IgG with lectins in distinguishing healthy women from patients with advanced endometriosis, as well as in differentiation between the group of healthy women and patients with non-endometriosis, was also analyzed by cluster analysis. For the analysis of the relative reactivities with the panel of lectins used, MPL, VVL, Jacalin and PHA-L meet the following criteria: they differentiate advanced endometriosis and non-endometriosis patients from the control group of healthy women as well as having high clinical value according to the results of ROC curve analysis (AUC = 1). The results obtained for a group of healthy subjects and women with advanced endometriosis are shown in Figure 5. The analysis was performed for 53 samples. The first cluster, distinguished at 53.2% distance (Cluster 1), consists of 22 samples, of which 19 were from the control group (100% of the control group) and 3 samples were from the advanced endometriosis group (4%). The next group, homogenous (Cluster 2 at 45.2% distance), was composed of advanced endometriosis samples only. Similarly, cluster analysis was performed for the non-endometriosis and control groups (Figure 6) for 51 samples. The first cluster could be separated at a distance of 55.2%. This group was homogenous and consisted of 32 non-endometriosis samples (100% of the group). Another homogenous group (Cluster 2), in which 19 samples (100%) were from the control group, could be separated at a 28% distance.

## 3. Discussion

Endometriosis is considered an autoimmune disease associated with a dysfunction of natural immunity, because it fulfills most of the classification criteria for such disorders. It is manifested by tissue damage, production of autoantibodies (against endometrium, ovary, phospholipids, and histones), and association with other autoimmune diseases [42]. In the present work, the profile and degree of O-glycosylation of human blood serum IgG were analyzed in the context of advanced endometriosis for the first time. 

The first aim of our study was to check whether, additionally to biantennary N-glycans, O-glycans and highly branched N-glycans are expressed in serum IgG in advanced endometriosis. Our intention was also to check whether there are any changes in the profile and degree of O-glycans’ exposition and the degree of expression of highly branched N-glycans in serum IgG, and whether the observed alterations, if present, may be used as an additional tool differentiating patients with advanced endometriosis from healthy women and patients with other gynecological diseases with accompanying inflammation. Moreover, we were also interested in whether the analysis of O-glycosylation profile and degree as well as the expression of multi-antennary N-glycans in native serum IgG (s-IgG), without prior IgG isolation, could be helpful in non-invasive diagnostics of advanced endometriosis. To our knowledge, this is the first analysis of such a type conducted in the context of advanced endometriosis diagnostics. 

It should be underlined that we were first to show the presence of O-glycans in blood serum IgG in women with advanced endometriosis. The results of our study showed that the relative reactivities of isolated serum IgG O-glycans with specific lectins were significantly higher in women with advanced endometriosis and those suffering from other gynecological diseases with an inflammatory background than in the group of healthy women. It is also worthy of attention that the relative reactivity of i-IgG O-glycans with Jacalin significantly differentiated the advanced endometriosis patients from women with other gynecological diseases, which was most probably caused by increased expression of core 3 type O-glycans in the group of non-endometriosis patients. Moreover, our study also showed that the relative reactivities of serum IgG with PHA-L lectin, specific to multi-antennary N-glycans, were significantly higher in advanced endometriosis and non-endometriosis patients than in the healthy control group, indicating elevated expression of multi-branched three and tetra-antennary N-glycans in the E and NE groups. The results of cluster analysis confirmed the usefulness of the determinations of relative reactivities of i-IgG glycans with MPL, VVL, Jacalin, and PHA-L for distinguishing healthy women from women with advanced endometriosis and women without endometriosis but suffering from other gynecological diseases.

The present study has shown that the values of the MPL/VVL ratio and the relative reactivities of isolated serum IgG O-glycans with MPL and VVL, which reflect the expression of T-antigen and Tn-antigen, respectively, were significantly higher in women with advanced endometriosis than in the healthy control group. We observed a similar relationship between these groups in the relative reactivities of i-IgG O-glycans with Jacalin. Moreover, in non-endometriosis patients, the relative reactivities of i-IgG glycans with lectins specific to O-linked oligosaccharides, as well as values of MPL/VVL ratio, were significantly higher than in the control group of healthy women. Interestingly, in comparison to the group of healthy women and patients with advanced endometriosis, the presence of significantly higher relative reactivities of isolated IgG with Jacalin was observed among non-endometriosis patients. The above interrelationship is especially important from a diagnostic point of view, because we were also looking for parameters that could differentiate advanced endometriosis from other gynecological diseases. Here these parameters appear to be the degree of expression of Jacalin-reactive core 3 O-glycans in i-IgG. To confirm our observations, additional studies on a more representative number of samples in each examined groups of patients should be provided. 

Jacalin is a lectin specific mainly to core 1 O-linked glycans, and binds O-glycosylated proteins. Although Jacalin’s preferences for sugars that are T/Tn antigens are well understood, its specificity has not been clarified in detail [43,44]. A study by Tachibana [45] documented that apart from high Jacalin affinity for the T-antigen and Tn-antigen, this lectin also showed a significant affinity for glycans composed of core 3 oligosaccharide structures and for sialo-T-antigen (ST), but it was unable to bind to glycans forming core 2, core 6 and sialo-Tn-antigen (STn) oligosaccharide structures. Based on the results of our previous study [36], in which we analyzed serum IgG sialylation in advanced endometriosis, we observed the presence of weak negative correlations between i-IgG glycans reactivity with Jacalin and their reactivity with sialo-specific lectins MAA and SNA (data not shown). This confirms that in the case of i-IgG, Jacalin reacts mainly with desialylated O-linked glycans, and additionally that the observed differences between non-endometriosis and advanced endometriosis groups may be caused by the expression of types of O-glycan structures other than core 1, most probably core 3, in IgG isolates. Another question is the availability of O-linked oligosaccharide structures for lectins, which, based on our observations, seems to be much greater when isolated protein is examined, especially when glycans are present in hard-to-reach places in the protein molecule.

In the case of s-IgG, the significantly higher expression of T-antigen was only observed in the non-endometriosis group when compared with advanced endometriosis patients and healthy women. However, these significances were weak. No other significant differences between examined groups were observed in relative reactivities of s-IgG O-glycans with lectins used. 

Our findings indicate that in advanced endometriosis and other gynecological diseases (NE group), the expression of T- and Tn-antigens in i-IgG increases in comparison to healthy women, which is most probably caused by the accompanied inflammatory condition rather than gynecological disease. It should be also mentioned that the differences between examined groups in the expression of O-glycans when native serum IgG was analyzed are not similar to those observed for i-IgG. As we mentioned above, this may be caused by insufficient availability of O-glycans for lectins in the native protein as compared to IgG isolated from biological material, which seems to be confirmed by the observed weak correlations, or lack thereof, between the relative reactivities of s-IgG and i-IgG O-glycans with specific lectins. Due to the variety of O-glycans’ functions in the human body, any changes in glycoproteins’ O-glycosylation profile and degree are important, as they are associated with the development of many diseases, including tumor progression [46,47], diabetes [40], and Alzheimer’s Disease [48,49]. IgG O-glycosylation, in contrast to N-glycosylation, is a poorly understood process. To date, little information is available about IgG O-glycosylation. The study provided by Plomp et al. [24] proved that there are O-glycans in the isolated serum IgG3. There are no reports on changes in IgG O-glycosylation pattern and/or degree in relation to pathological conditions. In our study on i-IgG, the expression of both T-antigen and Tn-antigen significantly increased in advanced endometriosis and non-endometriosis patients when compared with healthy women. 

MPL, VVL, and Jacalin may react with complete O-glycans as well as with their truncated form. However, they have different priorities of reactivity with each of these structures. The observed significant positive correlations between relative reactivities of MPL, VVL, and Jacalin with IgG O-glycans indicate that in patients with advanced endometriosis and other gynecological diseases, together with an increase of T-antigen expression, the expression of truncated forms of O-glycans is also higher in comparison to healthy women. The above interrelationships are especially visible for IgG isolates. 

Advanced endometriosis is often compared to cancer and is a known precursor to several types of ovarian cancer [50]. This could explain the significantly higher expression of PHA-L-reactive multi-antennary N-glycans in serum i-IgG in advanced endometriosis in comparison to healthy women, in whom multi-antennary N-glycans in i-IgG seem to be absent. On the other hand, in the group of non-endometriosis patients, i-IgG reactivities with PHA-L were the highest out of all three of the examined groups of women. Based on our previous examinations of serum IgG relative reactivities with sialo-specific lectins in advanced endometriosis [36], we observed the presence of weak negative correlations between i-IgG reactivities with PHA-L and its reactivities with MAA and SNA (data not shown). Taking the above observations into account, we can conclude that in advanced endometriosis and other gynecological diseases with an inflammatory background, the observed increase in expression of multi-antennary N-glycans in serum i-IgG is accompanied by decreased sialylation. However, considering that the sialo-specific lectins we used for terminal sialic acid detection do not recognize whether sialic acid is a part of N-glycans or O-glycans, structural studies should be carried out to unambiguously define the oligosaccharide composition of IgG N- and O-glycans in inflammatory diseases, including advanced endometriosis and other gynecological diseases. It should be mentioned that in glycosylation studies with lectin-ELISA, the obtained results do not reflect the exact structure of oligosaccharides but may indicate their potential bioavailability for specific ligands. Lectins may be capable of forming less favorable bonds, and steric hindrances resulting in restricted access to the glycoprotein, especially in the native biological sample, should also be taken into account. Nevertheless, the undoubted benefit of this type of examination is that the glycosylation analysis of glycoproteins present in a native biological fluid, using lectins specific to sugar structures, reflects the sugar-ligand reaction and actual availability of the analyzed glycoepitopes in the native microenvironment, and thus also their potential for in vivo interactions [36,38,39,51]. One of our research goals was to establish whether the analysis of IgG O-glycosylation directly in the serum, without prior protein isolation, would be sufficient to differentiate the group of patients with advanced endometriosis from other study groups, and the conducted research helped us answer this question. Considering the results of the analysis of the degree of serum IgG O-glycans and highly branched N-glycans’ expression presented above, the availability of oligosaccharides for the lectins used seems to be insufficient in the case of native IgG without its prior isolation.

To check the usefulness of the obtained results for the clinical differentiation of all three examined groups of women, ROC curve analysis was done. In the case of s-IgG, and only for non-endometriosis patients versus a control group of healthy women, a moderate clinical value for IgG MPL-reactive O-glycans expression was demonstrated (AUC = 0.737). For the remaining parameters, the clinical value was limited. The clinical usefulness of the examined parameters for the differentiation of women with advanced endometriosis from healthy women was not demonstrated. In the case of i-IgG, we observed a maximum high clinical value (AUC = 1) for IgG relative reactivities with all four lectins used, both when comparing women with advanced endometriosis to healthy women and healthy women to a non-endometriosis group. The results of ROC curve analysis obtained for the relative reactivities of i-IgG O-glycans with Jacalin in the E and NE groups showed its limited clinical utility (AUC = 0.661; *p* = 0.0106), with a sensitivity and specificity of 0.625 and 0.694, respectively (data not shown). The conducted cluster analysis confirmed the clinical usefulness of the analysis of i-IgG glycans’ relative reactivities with MPL, VVL, Jacalin, and PHA-L for the differentiation of healthy women from women with advanced endometriosis as well as women with gynecological diseases other than endometriosis. While our research has shown that both the expression of O-glycans and highly branched N-glycans in IgG may have a potential application in the diagnostics of advanced endometriosis, at the present stage of research, these conclusions mainly concern IgG isolated from serum. This makes it difficult to apply this type of determination in routine diagnostics due to the laborious and time-consuming procedure of protein isolation and purification. Nevertheless, this direction of research seems to be promising, and the development of a simple and fast protein isolation procedure would be very helpful.

## 4. Materials and Methods

### 4.1. Patient Samples

The study material was blood sera and IgG isolated from serum samples. The serum samples were derived from patients diagnosed with stages III and IV of endometriosis (E—advanced endometriosis; n = 34, mean age: 34.5 ± 7 years) and from women without endometriosis (NE—non-endometriosis; n = 32, mean age: 37.5 ± 8 years) were collected at the Department of Oncological Gynecology, Wroclaw Comprehensive Cancer Center, Poland. The study was conducted in agreement with the Helsinki II declaration and the protocol was approved by the Bioethics Human Research Committee of the Wroclaw Medical University (Permission No. KB-293/2016 and KB-719/2018). E and NE patients underwent surgical interventions, mainly laparoscopic, and after histological verification were classified to their proper groups. Women with advanced endometriosis were classified according to extent and severity of disease according to the revised American Fertility Society (rAFS) classification. Patients from the non-endometriosis group had histologically confirmed benign ovarian cysts with severe dysplasia—CIN 3 (cervical intraepithelial neoplasia grade 3) or leiomyomas. Additionally, at the Department of Laboratory Diagnostics, Wroclaw Medical University, serum samples from healthy female volunteers were collected (C—control group; n = 19, mean age: 40.3 ± 8 years, positive opinion of Bioethics Committee No KB-117/2020). The control group of healthy women had no symptoms or history connected with endometriosis, were non-pregnant, and did not suffer from any gynecological or inflammatory diseases. Serum samples examined in this study were collected from women on any day of the menstrual cycle. Before starting the study, all participants gave written and informed consent.

### 4.2. IgG Isolation

Immunoglobulin G was isolated from serum samples using affinity chromatography on the Protein A/Protein G Sepharose column, according to the procedure described previously by Ey et al. [52] and Sołkiewicz et al. [36]. In short: after 1:1 dilution in 50 mM TBS, pH 8.0, the serum sample (0.5 mL) was applied to the column (1 mL) and washed with the initial TBS solution. IgG was eluted from the column with 0.1 M glycine/HCl, pH 2.7 and immediately neutralized with 1 M Tris to avoid IgG degradation. The elution profile was determined from the absorbance measurement at 280 nm. An Amicon Ultra-15 centrifuge filter with an Ultracel-100 membrane (Millipore, Merck, Germany) was used to combine and concentrate the IgG-containing fractions. IgG concentration was determined spectrophotometrically on a polystyrene 96-well microtiter plate (Maxisorp, Dako, Denmark) using the bicinchoninic acid (BCA) colorimetric micro method [53]. The IgG solution was diluted with water when necessary, and 200 mL of a 50:1 mixture of stock solutions A and B was added to 10 mL of IgG solution. In the next step, the plate was incubated for 30 min at 37 °C. Bovine serum albumin (BSA) in concentrations of 0, 2, 4, 6, 8, and 10 μg/well was used as a standard. The absorbances were measured against a blank sample at 562 nm, and a standard curve was used to read the IgG concentrations, which were expressed in μg/mL [36]. 

### 4.3. Lectin-ELISA

The profile and degree of IgG O-glycosylation were determined using the previously described method [39,40]: modified solid-phase lectin-ELISA. The expression of O-glycans was studied with biotinylated lectins: MPL (*Maclura pomifera* lectin), VVL (*Vicia villosa* lectin) and Jacalin (*Artocarpus integrifolia* lectin). Additionally, PHA-L (*Phaseolus vulgaris* leucoagglutinin), selective for tri- and/or tetra-antennary N-linked glycans, was used [54] (Vector Laboratories Inc., Burlingame, CA, USA). For specificity of lectins see Table 6.

IgG concentrations in whole sera (s-IgG) and in IgG isolates (i-IgG), necessary for the calculation of IgG amount to lectin-ELISA, were determined previously using the turbidimetric method [58] as well as the BCA method [36] described above, respectively. The microtiter plates (Maxisorp, Dako, Denmark) were incubated with 0.01 mg/mL protein G (Abcam, Boston, MA, USA) solution in 10 mM TBS pH 7.4 for 2 h at 37 °C, then 4 °C overnight. The plates were then coated with native IgG diluted 10 mM TBS-T (TBS containing 0.1% Tween, pH 7.4) in an amount of 800 ng native IgG in 50 µL solution per well, and incubated for 3 h at 37 °C. For isolated IgG, the wells of microtiter plates were covered with 800 ng of isolated IgG diluted with 10 mM TBS, pH = 8.5 (total volume: 50 µL per well), and incubated for 24 h at 24 °C. After washing (TBS-T, pH = 7.5), the plates were incubated with biotinylated lectins for 60 min, 37 °C, which were diluted with 10 mM TBS-T as follows: MPL—1:1000, VVL—1:1000, Jacalin—1:5000 and PHA-L—1:250. Next, the plates were incubated with phosphatase-labeled ExtrAvidin (Sigma Chemical Co., St. Louis, MO, USA) for 30 min at 37°C. After the incubation, the phosphatase reaction was developed with a substrate, p-nitrophenyl phosphate. The reaction was stopped with 100 μL of 1 M NaOH per well and the absorbance was read at 405 nm, reference filter 630 nm, with a Mindray-96A microplate reader (Shenzhen Mindray Bio-Medical Electronics Co., Shenzhen, China). All samples were examined in duplicate. Background absorbances were measured for samples in which all reagents were present, but the biological sample was replaced with 10 mM TBS-T. Samples relative reactivities with lectins were expressed in absorbance units (AU).

### 4.4. Statistical Analysis

Statistical analysis was performed with the STATISTICA 13.3PL (StatSoft Polska Sp. z o.o., Warsaw, Poland). All results are presented as mean ± SD (standard deviation), and the differences between the groups were presented as bar graphs. According to a Shapiro–Wilk W test, the values did not fit a normal distribution, thus the nonparametric Mann–Whitney U test was used to determine differences among the groups. Correlations with a 95% confidence interval between examined parameters were tested by Spearman’s rank analysis. For the determination the strength of Spearman’s rank correlations the following classification was used: 0.0 ≤ r ≤ 0.2—lack of correlation; 0.2 < r ≤ 0.4—weak correlation; 0.4 < r ≤ 0.7—moderate correlation; 0.7 < r ≤ 0.9—strong correlation; 0.9 < r ≤ 1.0—very strong correlation. A two-tailed *p*-value of less than 0.05 was considered significant. The clinical value of determined parameters was analyzed using receiver operating characteristic (ROC) curves. Additionally, cluster analysis based on divisive hierarchical clustering was applied to estimate the diagnostic usefulness of measured parameters. For this analysis, the results are presented as a dendrogram, starting from one cluster in which all subjects (patients and controls) are gathered. In the next step, the subjects were clustered. Those with similar values of all analyzed traits were grouped together, while subjects with different ones formed a separate cluster. In summary, we observed that the greater the distance of separation, the greater were the differences in subject characteristics. Euclidean distance was used for similarity estimation. The scheme of statistical analysis of results obtained in the present study was adopted from our previous experience [36,38]. 

## 5. Conclusions

Endometriosis, due to non-specific symptoms, as well as the lack of sensitive and specific tests that would be available in routine clinical diagnostics, still remains in the group of late-diagnosed diseases. Therefore, it is extremely important to define a panel of diagnostic markers specific to this disease. However, the expression of serum IgG O-glycans is not very deeply explored. Our results indicate the presence of O-glycans in serum IgG in advanced endometriosis and other gynecological diseases, and changes in the profile and degree of serum IgG O-glycosylation between patients with gynecological diseases compared to the group of healthy women were also observed. Moreover, we have also shown that, except for the presence in IgG of typical biantennary N-glycans, highly branched N-glycans are also expressed, mainly in advanced endometriosis and non-endometriosis groups. Significantly higher expression of multi-antennary N-glycans in blood serum IgG in women with advanced endometriosis and with other gynecological diseases compared to healthy women clearly indicates that the presence of highly branched N-glycans is linked with pathological conditions accompanied by inflammation. The results obtained in our study have allowed for the selection of parameters helpful in the diagnostics of advanced endometriosis and may be used to direct future research in this field. We believe that the results of this study will also contribute to a better understanding of the molecular mechanisms accompanying this disease and facilitate the development of specific and sensitive diagnostic markers of advanced endometriosis in the future.

### 5.1. Strengths of the Study

1. We are the first to show the presence of O-glycans in blood serum IgG in women with advanced endometriosis.
2. The relative reactivities of isolated serum IgG O-glycans with specific lectins were significantly higher in women with advanced endometriosis and the group of women with gynecological diseases other than endometriosis in comparison to the group of healthy women.
3. The relative reactivity of i-IgG O-glycans with Jacalin significantly differentiates the advanced endometriosis patients from women with other gynecological diseases, which was most probably caused by increased expression of core 3 type O-glycans in the case of non-endometriosis patients.
4. The relative reactivities of blood serum IgG glycans with PHA-L in advanced endometriosis and non-endometriosis patients were significantly higher than in the control group of healthy women, showing increased expression of multi-branched three and tetra-antennary N-glycans in the E and NE groups.
5. Cluster analysis confirmed the usefulness of the determinations of relative reactivities of i-IgG glycans with MPL, VVL, Jacalin, and PHA-L for distinguishing healthy women from women with advanced endometriosis and women without endometriosis but suffering from other gynecological diseases.

### 5.2. Limitations of the Study

1. Significant differences in relative reactivities of IgG glycans with the lectins used were observed only for IgG isolates, which was most probably caused by better bioavailability of oligosaccharides for lectins when IgG was isolated from biological material. However, given that the process of isolation and purification of the protein is long and laborious, the differences observed between the studied groups in the relative reactivities of IgG glycans with lectins used should be treated as an additional cognitive aspect that could be difficult to apply in routine diagnostics.
2. The lack of women suffering from early stages of endometriosis made it impossible to check the utility of lectin-ELISA tests used in the present study for diagnostics of the early stages of disease development.

## Figures and Tables

**Table 1:** Relative reactivities of serum native IgG and isolated serum IgG.

	Relative Reactivity with Lectins (AU)									
Group	*MPL (s)*	*MPL (i)*	*VVL (s)*	*VVL (i)*	*MPL/VVL Ratio* *(s)*	*MPL/VVL Ratio* *(i)*	*Jacalin (s)*	*Jacalin (i)*	*PHA-L (s)*	*PHA-L (i)*
**E** **n = 34**	0.118 ± 0.045	0.342 ± 0.160	0.028 ± 0.031	0.061 ± 0.051	7.361 ± 5.705	7.465 ± 4.439	0.260 ± 0.076	1.025 ± 0.094	0.197 ± 0.235	0.340 ± 0.148
**NE** **n = 32**	0.169 ± 0.111 *p^E^* = 0.022764	0.310 ± 0.115	0.047 ± 0.076	0.080 ± 0.097	6.228 ± 4.742	5.885 ± 3.383	0.244 ± 0.082	1.103 ± 0.138 *p^E^* = 0.006401	0.263 ± 0.270	0.402 ± 0.169
**C** **n = 19**	0.103 ± 0.042 *p^NE^* = 0.005500	0.020 ± 0.025 *p^E^* = 0.000000 *p^NE^* = 0.000000	0.029 ± 0.022	0.001 ± 0.002 *p^E^* = 0.000000 *p^NE^* = 0.000000	6.227 ± 6.382	2.750 ± 5.546 *p^E^* = 0.000000 *p^NE^* = 0.000317	0.246 ± 0.084	0.106 ± 0.140 *p^E^* = 0.000000 *p^NE^* = 0.000000	0.180 ± 0.123	0.002 ± 0.006 *p^E^* = 0.000000 *p^NE^* = 0.000000

Significant differences versus groups: E with advanced endometriosis (E), NE non-endometriosis (NE), C—control group of healthy women. s—serum native IgG, i—isolated serum IgG. For specificity of lectins see Materials and Methods section. Significant differences were accepted for a *p*-value of less than 0.05.

**Table 2:** Correlations between relative reactivities of s-IgG and i-IgG O-glycans with specific lectins.

	s-IgG	i-IgG
Correlations between IgG Relative Reactivities with Lectins	Spearman Rank Coefficient (r)	Spearman Rank Coefficient (r)
**MPL vs. VVL**	0.566 *p* = 0.000000	0.737 *p* = 0.000000
**MPL vs. Jacalin**	0.549 *p* = 0.000000	0.832 *p* = 0.000000
**VVL vs. Jacalin**	0.247 *p* = 0.000000	0.666 *p* = 0.000000

Serum native IgG—s-IgG, serum IgG isolates—i-IgG. MPL—*Maclura pomifera* lectin, VVL—*Vicia villosa* lectin, Jacalin—*Artocarpus integrifolia* lectin. A two-tailed *p*-value of less than 0.05 was considered significant. For specificity of lectins see Materials and Methods section.

**Table 3:** Correlations of relative reactivities of s-IgG and i-IgG glycans with lectins.

Correlations between Relative Reactivity of Lectins with s-IgG vs. i-IgG	Spearman Rank Coefficient (r)
MPL (s) vs. MPL (i)	0.260874 *p* = 0.015890
VVL (s) vs. VVL (i)	
MPL/VVL (s) vs. MPL/VVL (i)	0.255850 *p* = 0.018109
Jacalin (s) vs. Jacalin (i)	
PHA-L (s) vs. PHA-L (i)	0.279398 *p* = 0.009610

Serum native IgG—s-IgG, serum IgG isolates—i-IgG. MPL—Maclura pomifera lectin, VVL—Vicia villosa lectin, MPL/VVL—O-glycosylation ratio, Jacalin—Artocarpus integrifolia lectin and PHA-L—Phaseolus vulgaris leucoagglutinin. A two-tailed p-value of less than 0.05 was considered significant. For specificity of lectins see Materials and Methods section.

**Table 4:** Values of ROC curve analysis of s-IgG and i-IgG glycans’ relative reactivities with lectins for women with advanced endometriosis and healthy subjects.

Lectin	AUC	AUC with 95% Confidence Interval	Cut-Off Point	Sensitivity	Specificity	*p*
**s-IgG**						
**MPL**	0.576	0.414–0.738	0.134	0.353	0.842	0.3580
**VVL**	0.561	0.393–0.73	0.018	0.559	0.632	0.4771
**MPL/VVL**	0.611	0.479–0.744	5.265	0.647	0.684	0.1001
**Jacalin**	0.565	0.406–0.724	0.324	0.265	0.947	0.4242
**PHA-L**	0.406	0.254–0.557	0.348	0.265	0.947	0.2215
**i-IgG**						
**MPL**	**1**	1–1	0.085	1.000	1.000	0.0000
**VVL**	**1**	1–1	0.01	1.000	1.000	0.0000
**MPL/VVL**	**0.811**	0.693–0.929	1.854	1.000	0.789	0.0000
**Jacalin**	**1**	1–1	0.852	1.000	1.000	0.0000
**PHA-L**	**1**	1–1	0.102	1.000	1.000	0.0000

MPL—*Maclura pomifera* lectin, VVL—*Vicia villosa* lectin, MPL/VVL—O-glycosylation ratio, Jacalin—*Artocarpus integrifolia* lectin and PHA-L—*Phaseolus vulgaris* leucoagglutinin. For specificity of lectins see Materials and Methods section. The analysis was performed for patients with advanced endometriosis and a control group of healthy women. Clinical utility, based on AUC value, can be defined as: 0–0.5—zero, 0.5–0.7—limited, 0.7–0.9—moderate, and >0.9—high. A two-tailed *p*-value of less than 0.05 was considered significant.

**Table 5:** ROC curve analysis of s-IgG and i-IgG glycans’ relative reactivities with specific lectins for non-endometriosis and healthy subjects.

Lectin	AUC	AUC with 95% Confidence Interval	Cut-Off Point	Sensitivity	Specificity	*p*
**s-IgG**						
**MPL**	0.737	0.601–0.873	0.118	0.656	0.737	0.0006
**VVL**	0.544	0.378–0.711	0.035	0.469	0.737	0.6011
**MPL/VVL**	0.582	0.412–0.753	3.48	0.813	0.421	0.3450
**Jacalin**	0.523	0.36–0.686	0.193	0.344	0.789	0.7823
**PHA-L**	0.546	0.388–0.704	0.23	0.469	0.789	0.5669
**i-IgG**						
**MPL**	**1**	1–1	0.126	1.000	1.000	0.0000
**VVL**	**1**	1–1	0.015	1.000	1.000	0.0000
**MPL/VVL**	**0.801**	0.627–0.975	0.975	1.000	0.789	0.0007
**Jacalin**	**1**	1–1	0.72	1.000	1.000	0.0000
**PHA-L**	**1**	1–1	0.166	1.000	1.000	0.0000

MPL—*Maclura pomifera* lectin, VVL—*Vicia villosa* lectin, MPL/VVL—O-glycosylation ratio, Jacalin—*Artocarpus integrifolia* lectin and PHA-L—*Phaseolus vulgaris* leucoagglutinin. For specificity of lectins see Materials and Methods section. The analysis was performed for non-endometriosis and a control group of healthy women. A two-tailed *p*-value of less than 0.05 was considered significant. Clinical utility, based on AUC value, can be defined as: 0–0.5—zero, 0.5–0.7—limited, 0.7–0.9—moderate, and >0.9—high.

**Table 6:** Specificity of the lectins used in the study.

Lectin Source	Specificity for Sugar Structures
MPL (*Maclura pomifera* lectin)	T (Galβ1,3GalNAc) and Tn antigen (single GalNAc) [55]
VVL (*Vicia villosa* lectin)	Tn antigen (single GalNAc) [56,57]
Jacalin (*Artocarpus integrifolia* lectin)	T antigen (Galβ1,3GalNAc), Tn antigen (single GalNAc), sTn antigen (NeuAcα2,6GalNAc) [45]
PHA-L (*Phaseolus vulgaris* lectin)	tri/tetra-antennary N-glycans, binds to β1,6 branches of tri- and tetra-antennary oligosaccharides [54]

Gal—galactose; GalNAc—N-Acetylgalactosamine; NeuAc—N-Acetylneuraminic Acid.

## Data Availability

Not applicable.
